# Assessing Noise Levels in a Mexican Neonatal Intensive Care Unit: Results From an Observational Study

**DOI:** 10.1155/nrp/9197371

**Published:** 2025-09-18

**Authors:** Yari Jaguey-Hernández, Claudia Atala Trejo-García, Karina Isabel Casco-Gallardo, Sheila Adriana Mendoza-Mojica, José Antonio Guerrero-Solano

**Affiliations:** ^1^Department of Agroindustrial Engineering, Polytechnic University of Francisco I. Madero, Tepatepec, Hidalgo, Mexico; ^2^Academic Area of Nursing, Graduate School of Tlahuelilpan, Autonomous University of the State of Hidalgo, Pachuca, Hidalgo, Mexico

**Keywords:** decibels, hospital, neonatal intensive care unit, newborns, NICU, noise metrics, noise source

## Abstract

**Background:** In recent decades, noise pollution has become a significant concern, especially in sensitive environments such as hospitals. For neonates in neonatal intensive care units (NICUs), noise can have serious implications due to their underdeveloped auditory and physiological systems. Elevated noise levels in NICUs have been linked to disruptions in normal physiological parameters and can negatively impact both neonatal health and staff performance.

**Aim:** To identify and quantify sources of noise in the intensive care unit of a hospital NICU.

**Methods:** An observational study was conducted to assess noise levels in a Mexican NICU. Noise measurements were taken across 22 different scenarios identified as potential noise sources, during all three shifts to capture variations throughout the day.

**Results:** Noise levels ranged from 45 to 70 decibels (dB). The nursing shift change was identified as the scenario with the highest noise level, while the paper dispenser area recorded the lowest. These findings highlight specific times and locations within the NICU where noise reduction interventions should be focused.

**Conclusions:** Targeted noise control strategies, especially during shift changes, could significantly improve the acoustic environment in NICUs, helping to safeguard neonatal well-being and staff performance.

## 1. Introduction

Noise pollution is defined as an unwanted, unpleasant, or disruptive acoustic disturbance that can interfere with communication, comfort, or health [1]. The intensity of noise is particularly important, as high levels have been shown to be detrimental to human health. Consequently, regulations have been established to control noise levels, though these vary internationally [2]. The World Health Organization (WHO) recommends that the equivalent continuous sound pressure level (Leq) should not exceed 40 decibels (dB) during the night and 65 dB during the day to be considered acceptable [3]. In hospital settings, the WHO advises maintaining sound levels below 35 dB during the day and 30 dB at night [4, 5].

Hospitals are environments of particular concern regarding noise levels, as they conduct numerous activities aimed at restoring patient health. A key area of interest is the neonatal intensive care unit (NICU), which houses newborns undergoing various diagnostic and therapeutic procedures to address serious health conditions threatening their survival or development. These include prematurity, bronchopulmonary dysplasia, congenital cardiovascular anomalies, necrotizing enterocolitis, infections, and neurological complications [6], among others. These patients require comprehensive medical support including continuous monitoring of vital signs, administration of pharmacologic therapies, mechanical respiratory assistance, nutritional support, and interventions aimed at promoting neurosensory, motor, and cognitive development.

The NICU serves as a substitute for the intrauterine environment [7], where the mother's body and amniotic fluid naturally attenuate high noise levels [8]. To safeguard neonates from excessive noise exposure, the American Academy of Pediatrics (AAP) recommends maintaining noise levels at or below 35 dB during nighttime and up to 45 dB during daytime, with a maximum sound level (*L*_max_) below 60 dB [9, 10].

The detrimental effects of noise on health have been thoroughly investigated. In neonates, whose nervous, immune, and auditory systems are still immature, exposure to excessive noise can adversely affect their health. Consequences include irritability, sleep disturbances, delayed speech development, behavioral issues, and, in severe cases, intraventricular hemorrhage [11–13]. These outcomes depend on factors such as noise intensity, duration, frequency, and individual susceptibility.

Elevated noise levels constitute a significant stressor for neonates [11]. Increased cortisol levels resulting from noise exposure have been linked to alterations in heart rate, manifesting as bradycardia or tachycardia, apnea, hypertension, decreased perfusion, and hypoxemia [12, 14]. These physiological stress responses can intensify oxygen consumption and reduce the caloric resources necessary for optimal growth and neurodevelopment [14–18].

Furthermore, noise may be perceived as a threat, inducing anxiety that interferes with concentration and cognitive functions. This effect can impair memory, learning, and decision-making processes, leading to chronic stress [19]. Elevated noise levels thus not only impact patients but are also correlated with increased rates of errors and accidents and decreased performance among hospital staff [17, 19].

This study aimed to identify and quantify sources of noise within a Level III NICU in a Mexican hospital to develop informed noise reduction strategies tailored for such critical care environments.

## 2. Materials and Methods

### 2.1. Study Design

This was a prospective, descriptive observational study conducted at a single hospital center, following the structure and recommendations of the Strengthening the Reporting of Observational Studies in Epidemiology (STROBE) guidelines to ensure transparency and quality in reporting observational epidemiological studies [20]. The research focused on describing and quantifying noise levels in a neonatal intensive care setting, with measurements systematically taken during different shifts, minimizing potential biases through replicated measurements and a standardized methodology. The details are provided in the following.

A nonprobabilistic convenience sampling was used, considering neonates admitted to the NICU during this period. Premature neonates born before 37 weeks of gestation, of both sexes, who were admitted to the NICU and whose parents or guardians signed informed consent were included. Exclusion criteria comprised neonates under analgesia, those whose parents or guardians did not sign informed consent, those in critical condition preventing proximity, and healthy neonates in the growth and development area. Elimination criteria included patient death during the study, neonates discharged during the study, neonates isolated due to infectious disease, and neonates with congenital malformations.

Noise levels were measured in the open-bay NICU room at Tula Hospital, located in Hidalgo, Mexico, between June and August 2024. The NICU has a capacity of 14 patients and operates on a three-shift schedule: morning shifts from 7:00 a.m. to 2:00 p.m., afternoon shifts from 2:00 p.m. to 8:00 p.m., and night shifts from 8:00 p.m. to 7:00 a.m.

A literature review was conducted to identify common sources of noise reported in NICUs. The methodology proposed by Capriolo et al. [21], with some modifications, was used to measure noise. Measurements were taken near the neonates at a distance of 30 cm, approximately at the infant's ear level, to accurately reflect their auditory environment using a portable sound level meter (Mengshen MS-M80A, Wuhan, China) capable of detecting levels ranging from 30 to 130 dB.

### 2.2. Measurement Instrument and Procedures

The measurements consisted of brief 2-min “snapshots” of noise from previously identified objects and events. Data collection was conducted during routine care within each of the three daily shifts in two distinct time periods. The initial assessment included measurements taken over 7 days, 7 evenings, and 7 nights. This sequence was repeated to ensure consistency and reliability of the measurements.

### 2.3. Data Collection and Analysis

The minimum sound level and *L*_max_ of each noise measurement were recorded. The data were stored in a database for subsequent statistical analysis using GraphPad Prism software. A one-way ANOVA followed by Tukey's post hoc test was applied, with *p* < 0.05 considered as statistically significant.

### 2.4. Ethics Statement

The study was conducted in accordance with the Declaration of Helsinki and the General Health Law on Research in Mexico. Although no identifiable patient or healthcare staff information was collected and the study did not meet the criteria for human subject research, it was approved by the Institutional Review Board of Tula General Hospital (registration number: HGT24-004).

## 3. Results

**Figure 1:**
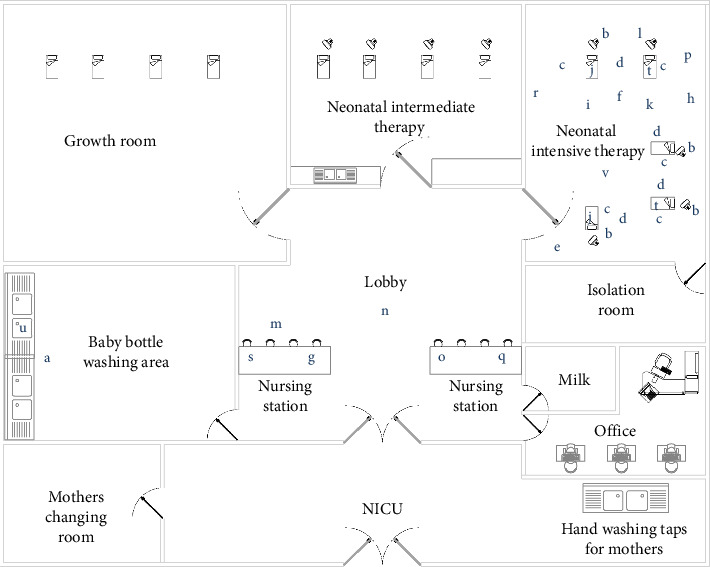
Spatial layout of the NICU showing designated areas. Blue letters indicate the specific noise sources identified and the precise locations where sound level measurements were taken using a sound level meter. Measurements were conducted in triplicate at various intensive care unit beds and common areas to ensure accuracy. Each letter corresponds to a distinct noise source as referenced in the study.

Three relevant articles were selected to support the identification of noise sources commonly reported in NICUs [22–24]. Twenty-two distinct situations and objects were identified as noise sources within the NICU under study, including (a) bottle collisions, (b) mechanical ventilator alarms, (c) incubator alarms, (d) curtain shifts, (e) side door movement, (f) Perfusor alarms, (g) room telephone, (h) opening or closing drawers of the red emergency cart, (i) nebulizer, (j) patient crying, (k) objects falling to the floor, (l) monitor alarms, (m) staff conversations, (n) nursing shift handover, (o) placement of objects at the nursing station, (p) placement of objects in the incubator, (q) paper dispensing, (r) placement of objects on the Pasteur table, (s) dragging of tables, (t) sucking, (u) water flow, and (v) medical visits (Figure 1). We evaluated noise levels in an open-bay NICU room, where higher noise levels were expected compared to single-room setups.

Of the 14 preterm infants admitted to the NICU, 5 did not meet the inclusion criteria. Consequently, the remaining 9 preterm infants who met the inclusion criteria and whose parents provided informed consent were included in the study. Noise measurements were taken in triplicate at a distance of 30 cm from each incubator.

Figure 1 illustrates the spatial configuration of the NICU, highlighting key areas such as the nurse central station and the bottle washing station, which were identified as significant contributors to noise. The neonatal intermediate therapy area accommodates four incubators. The neonatal intensive therapy area can house up to five incubators, each equipped with a cardiac monitor for continuous vital sign monitoring. Additional equipment, such as ventilators, infusion pumps, and therapy lamps, may also be present depending on patient needs.

The highest recorded noise level was 70.43 dB during nursing shift handovers, while the lowest was 44.83 dB during dispensing paper.  Table 1 summarizes the maximum, minimum, and average noise levels for 22 events per shift.

**Figure 2:**
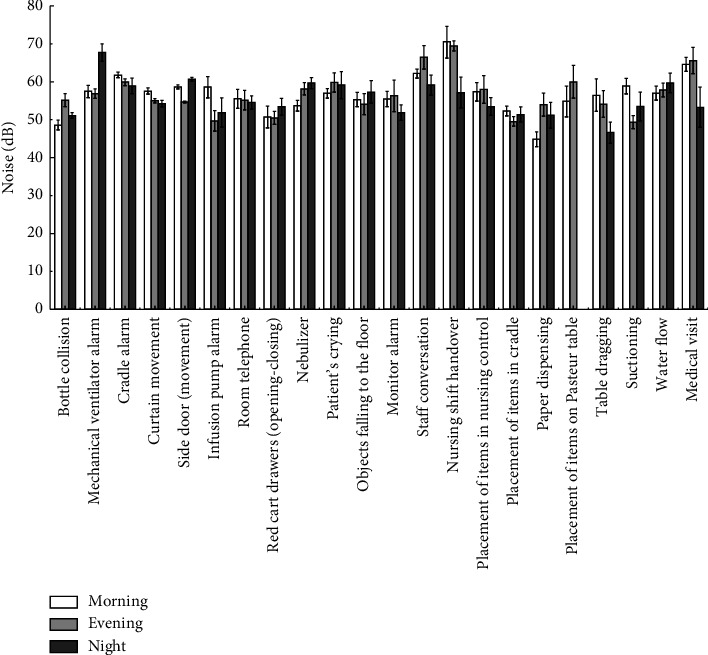
Noise level across 22 different NICU events by work shift.

The highest average noise level was observed during nursing shift handovers, reaching 65.67 dB, followed by staff conversations at 62.57 dB and medical visits at 61.14 dB. In contrast, the quietest activities included paper handling (50.00 dB), placing objects in the incubator (51.06 dB), opening or closing the drawers of the red emergency cart (51.52 dB), and bottle washing (51.59 dB) (Figure 2). Noise levels associated with equipment alarms were recorded as 53.37 dB for infusion pump alarms, 60.19 dB for crib alarms, and 60.67 dB for mechanical ventilator alarms. Notably, all measured noise levels exceeded the AAP recommended limits by more than 5 dB [10].

**Figure 3:**
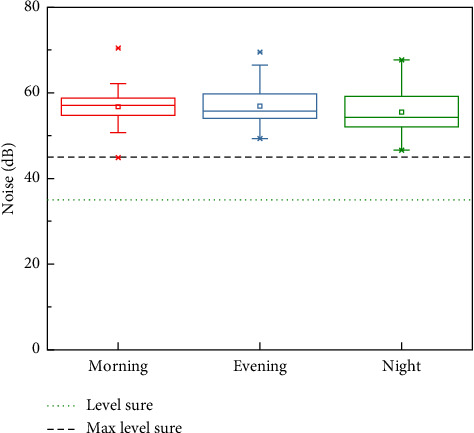
Comparison of average noise levels across different shifts.

Noise levels during shift changes were significantly higher than during regular working hours, with the morning shift being the noisiest and the night shift the quietest. A statistically significant difference (*p* < 0.05) in noise levels was observed during shift handovers, with lower levels during the night shift (Figure 3).

No significant differences (*p* < 0.05) were observed across shifts for noise generated by crib alarms, curtain movement, phone ringing, red cart opening and closing, patient crying, objects falling, monitor alarms, or object placement at the nursing station or in the crib.

## 4. Discussion

The NICU environment is characterized by prolonged separation of neonates from their parents and a lack of sensory-environmental support, compounded by potentially painful medical procedures. The absence of adequate auditory stimulation, coupled with restricted speech and language exposure, can negatively affect the auditory cortex of infants [1]. Although regulations often aim to achieve near silence in NICUs, this environment can inadvertently lead to sensory deprivation. Furthermore, certain sounds, such as parental voices, have been shown to positively stimulate neonatal development [15].

Previous studies have documented a wide range of noise levels in NICUs, from 36 to 120 dB [9, 25]. Exposure to noise levels exceeding 70 dB can contribute to cumulative hearing loss, while levels above 120 dB may result in acute hearing loss [26]. The highest noise level recorded in this study occurred during nursing shift changes (70.43 dB), which is lower than the maximum levels reported for Level IV NICUs by Mayhew et al. [27] (85 dB) and Capriolo et al. [21]. Nevertheless, this underscores the continuing need for effective noise management. Consistent with the findings by Khowaja et al. [11], the nursing shift change period involves the simultaneous presence of both outgoing and incoming staff. These transitions necessitate the exchange of critical patient information, which is essential to ensuring continuity of treatment, as well as patient safety and well-being [28]. Although elevated, the measured noise level remains below the levels reported in Level IV NICUs by Mayhew et al. and Capriolo et al. [21, 27], reaffirming the importance of noise control in neonatal care environments.

The primary sources of noise in the NICU include hospital equipment such as vital signs monitors and ventilators [14], infusion pumps, and staff activities such as conversations [21], equipment handling, and cleaning [12]. External environmental noise emanates from hallways, reception areas, equipment sounds, telephones, crying, and conversations among staff and family members [12, 29], as well as emergency alarms and sirens, all of which contribute to the overall noise levels in the NICU. Other significant noise sources include ventilation and air conditioning systems, and occasionally, construction and remodeling activities in hospital areas that may lack adequate acoustic insulation [30].

The observed noise levels in this study were similar to those reported by Rodríguez-Montaño et al. [12], who documented overall noise levels around 56 dB. Specifically, in our study, noise levels registered 56.76 dB in the morning, 56.78 dB in the afternoon, and 52.98 dB during the night shift. These data align with Mayhew et al. [27], who reported noise levels of 58.2 dB (morning), 53.6 dB (afternoon), and 54.5 dB (night). Notably, our lowest noise levels were recorded during the night shift, in contrast to Mayhew et al., who found the afternoon shift to be the quietest.

Noise measurements included maximum, minimum, and average values obtained from 22 events per shift, accounting for intrinsic sources within the hospital environment. Remarkably, 21 out of the 22 evaluated situations consistently exceeded the recommended noise limit of 45 dB set by the WHO [5] and the AAP [10, 31].

The highest noise levels were observed during nursing shift handovers, consistent with findings by Khowaja et al. [11]. These shift changes involve a greater number of personnel present in the NICU simultaneously, temporarily increasing the patient-to-nurse ratio and, consequently, noise levels. Visiting hours also contribute by increasing foot traffic and ambient noise.

To mitigate noise, the study recommends continuous staff training to raise awareness of associated health risks [19, 24]. It is advisable that shift handovers be conducted at least 2 m away from patients [1], and that only one staff member at a time approaches the patient to assess their condition. While equipment alarms remain essential for patient safety, their noise impact could be reduced by managing them via sensors placed at the nursing station, minimizing proximity noise near neonates.

- Noise levels from equipment alarms in this study were lower than those reported by Mayhew et al. [27]. For example, infusion pump alarms measured 53.37 dB here, compared to the 60.2–75 dB range reported previously. Similarly, noise from objects falling on the floor registered 55.59 dB in this study, which is approximately 25 dB lower than the values previously documented [27]. The activities with the lowest associated noise levels included paper handling, object placement, and bottle washing, suggesting that further reductions may be achievable through adjustments in practices and equipment use.

Incubators can amplify noise, with internal sound levels ranging from 56 to 62.1 dB [12, 32]. This underscores the necessity of implementing strategies to reduce noise transmission, including enhanced architectural design [1] and the use of auditory protection devices that attenuate high-frequency noises while permitting low-frequency sounds, such as human speech, to be heard [25]. Silicone earplugs have been demonstrated to improve the Mental Developmental Index in premature infants; however, they do not have a significant effect on the Psychomotor Developmental Index [18]. Moreover, their use is conditional and supported by low-quality evidence according to the Society of Critical Care Medicine Clinical Practice Guidelines [13].

Music therapy has also proven effective in lowering noise levels while positively influencing neurological development, parent–child bonding, and vital signs [1]. Additional noise reduction strategies include scheduling designated quiet periods, appointing noise supervisors, and conducting medical rounds away from patient areas [1, 27, 31].

Despite the high level of awareness among nursing staff about the detrimental effects of excessive noise [33], current interventions such as visual feedback warning systems have demonstrated effectiveness only in the short term [1, 34]. Although noise levels recorded in this study consistently exceeded the recommendations of the WHO and the AAP, they remained below thresholds linked to acute auditory trauma.

### 4.1. Limitations

This study has limitations. First, data were collected in a single NICU, which may restrict the generalizability of the findings to other settings with different infrastructure, staffing patterns, or clinical practices. Although noise measurements were conducted during routine care across various scenarios, the study did not include physiological analyses such as monitoring changes in oxygen saturation or heart rate, which could have provided further insight into the potential clinical impact of noise. Nonetheless, the results emphasize the urgent need to enhance the implementation of noise reduction strategies in NICUs to foster a more developmentally supportive environment for preterm and critically ill infants.

## 5. Conclusion

Excessive noise in NICUs presents a significant risk to neonatal recovery and neurodevelopment. This study underscores the importance of regular training for NICU staff aimed at noise reduction through practical measures such as speaking softly, keeping phones on silent mode, closing incubator doors gently, avoiding placing objects on incubators, and minimizing other environmental disturbances. In addition, ensuring that neonates receive 2–3 h of uninterrupted rest is vital to prevent overstimulation.

The findings reveal that noise levels in NICUs consistently exceed the limits recommended by the WHO and the AAP, highlighting the urgent need to revise current guidelines to better reflect contemporary clinical settings.

Effective noise management demands a multidisciplinary approach involving clinical staff, families, administrators, and design professionals. Key strategies include incorporating enhanced acoustic design, deploying low-noise equipment, and adjusting protocols during peak activity times such as shift handovers and medical rounds. Such a coordinated effort is essential to foster a healing environment that supports both neonatal health and staff well-being.

## Figures and Tables

**Table 1:** Summary of noise levels _(min, max, and average)_ for 22 NICU scenarios.

Sound variables		Morning		Evening		Night		Total average
		Range *L*_min_ − *L*_max_	*L*_average_	Range *L*_min_ − *L*_max_	*L*_average_	Range *L*_min_ − *L*_max_	*L*_average_	
a	Bottle collision	52–62	48.56 ± 2.74	50–62	55.11 ± 3.62	48–53	51.11 ± 1.69	51.59 ± 3.85
b	Mechanical ventilator alarm	52–61	57.44 ± 3.32	53–61	56.89 ± 2.62	58–72	67.67 ± 4.90	60.67 ± 6.20
c	Incubator alarm	60–64	61.78 ± 1.39	58–64	59.89 ± 1.90	55–64	58.89 ± 4.34	60.19 ± 3.00
d	Curtain shift	56–60	57.50 ± 1.91	53–56	55.00 ± 1.41	52–56	54.25 ± 2.06	55.58 ± 2.19
e	Side door movement	58–60	58.67 ± 1.15	54–55	54.67 ± 0.58	60–62	60.67 ± 1.15	58.00 ± 2.78
f	Perfusor alarm	49–68	58.56 ± 5.96	44–60	49.67 ± 5.74	40–63	51.89 ± 8.18	53.37 ± 7.51
g	Room phone	48–63	55.50 ± 5.43	50–64	55.17 ± 5.67	48–59	54.50 ± 3.83	55.06 ± 4.76
h	Opening or closing the drawers of the red emergency car	46–64	50.71 ± 6.24	44–46	50.43 ± 3.82	48–58	53.43 ± 4.58	51.52 ± 4.93
i	Nebulizer	48–58	53.63 ± 3.02	55–62	58.13 ± 3.36	55–64	59.63 ± 3.07	57.13 ± 3.98
j	Patient crying	54–62	57.00 ± 2.65	53–70	59.78 ± 5.43	53–73	59.11 ± 7.42	58.63 ± 5.44
k	Objects falling on the floor	49–62	55.33 ± 3.97	44–60	54.11 ± 5.82	50–70	57.33 ± 6.16	55.59 ± 5.37
l	Monitor alarm	49–62	55.43 ± 4.31	46–68	56.29 ± 8.85	46–60	51.86 ± 4.41	54.52 ± 6.23
m	Staff conversation	58–64	62.14 ± 2.48	60–76	66.43 ± 6.68	50–64	59.14 ± 5.76	62.57 ± 5.88
n	Nursing shift handover	61–89	70.43 ± 8.96	67–75	69.43 ± 2.88	46–75	57.14 ± 8.78	65.67 ± 9.38
o	Placement of objects at the nursing station	50–64	57.33 ± 5.12	50–71	58.00 ± 7.76	48–65	53.44 ± 5.00	56.26 ± 6.31
p	Placement of objects in the incubator	49–57	52.33 ± 2.88	46–53	49.50 ± 2.74	49–58	51.33 ± 4.37	51.06 ± 3.42
q	Dispensing of paper	40–50	44.83 ± 4.31	50–62	54.00 ± 6.57	44–58	51.17 ± 7.33	50.00 ± 7.04
r	Placement of objects on the Pasteur table	40–64	54.78 ± 4.03	49–71	60.00 ± 4.35	ND	ND	—
s	Dragging of tables	44–66	56.44 ± 4.31	43–68	54.11 ± 3.49	40–59	46.56 ± 2.85	52.37 ± 4.76
t	Sucking	54–60	58.83 ± 2.04	46–56	49.33 ± 1.70	42–64	53.50 ± 3.74	53.89 ± 8.51
u	Water flow	55–60	57.00 ± 1.78	52–64	57.83 ± 1.83	49–64	59.67 ± 2.54	58.17 ± 4.44
v	Medical visits	58–69	64.57 ± 1.79	56–73	65.57 ± 3.39	40–65	53.29 ± 5.28	61.14 ± 9.60
Average per turn		56.76 ± 2.02		56.79 ± 2.26		52.98 ± 2.52		

*Note:* Data are presented as mean standard deviation. *L*_min_, minimum sound level; *L*_max_, maximum sound level; *L*_average_, average sound level.

Abbreviation: ND, not detected.

## Data Availability

The data that support the findings of this study are available from the corresponding author upon reasonable request.
